# Development of a 32-gene signature using machine learning for accurate prediction of inflammatory bowel disease

**DOI:** 10.1186/s13619-022-00143-6

**Published:** 2023-01-05

**Authors:** Shicheng Yu, Mengxian Zhang, Zhaofeng Ye, Yalong Wang, Xu Wang, Ye-Guang Chen

**Affiliations:** 1 Guangzhou Institutes of Biomedicine and Health, Chinese Academy of Sciences, 190 Kaiyuan Avenue, Guangzhou Science Park, Luogang District, Guangzhou, 510530 China; 2 Guangzhou Laboratory, Guangzhou, 510700 China; 3 The State Key Laboratory of Membrane Biology, Tsinghua-Peking Center for Life Sciences, School of Life Sciences, Tsinghua University, Beijing, 100084 China; 4 School of Medicine, Tsinghua University, Beijing, 100084 China; 5 School of Basic Medicine, Nanchang University, Nanchang, 330031 China

**Keywords:** IBD, XGBoost, Signature genes, AI prediction, Biomarker

## Abstract

Inflammatory bowel disease (IBD) is a chronic inflammatory condition caused by multiple genetic and environmental factors. Numerous genes are implicated in the etiology of IBD, but the diagnosis of IBD is challenging. Here, XGBoost, a machine learning prediction model, has been used to distinguish IBD from healthy cases following elaborative feature selection. Using combined unsupervised clustering analysis and the XGBoost feature selection method, we successfully identified a 32-gene signature that can predict IBD occurrence in new cohorts with 0.8651 accuracy. The signature shows enrichment in neutrophil extracellular trap formation and cytokine signaling in the immune system. The probability threshold of the XGBoost-based classification model can be adjusted to fit personalized lifestyle and health status. Therefore, this study reveals potential IBD-related biomarkers that facilitate an effective personalized diagnosis of IBD.

**Supplementary Information:**

The online version contains supplementary material available at 10.1186/s13619-022-00143-6.

## Background

Inflammatory bowel disease (IBD), including ulcerative colitis (UC) and Crohn's disease (CD), is an idiopathic inflammatory bowel disease that heavily interferes with people’s quality of life (Rutgeerts et al. 2009). Ulcerative colitis unfolds the continuous inflammation of the colonic mucosa and submucosa, which usually involves the rectum first and gradually spreads to the whole colon, while Crohn's disease can involve the whole digestive tract, which is a discontinuous full-thickness inflammation, most often involving the terminal ileum, colon and perianal area (Feagins et al. 2011, Ordas et al. 2012). IBD patients are usually accompanied by disrupted stem cell dynamics and impaired epithelial regeneration capacity (Krishnan et al. 2011, Olafsson et al. 2020). Chronic inflammation can proceed to irreversible tissue destruction unless appropriate therapy is provided (Hosseinkhani et al. 2020). While the first step to treat IBD is to ease the pain, reduce inflammation, and facilitate tissue repair and regeneration (De Vry et al. 2007). Clinical data combined with mouse colitis models reveals that many important signaling pathways promote mucosal regeneration (Bergstrom et al. 2016, Han et al. 2020, He et al. 2018). For example, protease-activated receptor 2 (PAR2) signaling can mediate colonic mucosal regeneration through the stabilization of YAP (He et al. 2018). Clinical trials with consideration of mucosal regeneration and immune modulation may have promising results in treating IBD (Pak et al. 2018).

Genetic, microbial, environmental, and immunoregulatory factors have been suggested to contribute to IBD, but the exact cause of which is still unknown (Graham and Xavier 2020, Kiesslich et al. 2012). Thus, the identification of cellular and molecular mechanisms that contribute to different subtypes and developing phases of IBD is essential for developing targeted therapies (Eftychi et al. 2019, Matsukawa et al. 2016). Differentially expressed genes and pathways have been identified between inflamed and healthy control tissues of IBD patients, such as NF-κB, TNF-α, immune response, proinflammatory cytokines, and chemokines (Allen et al. 2012, Gadaleta et al. 2011). However, till now there are no ideal biological markers for IBD due to the complex genetic background and environmental factors (Khaki-Khatibi et al. 2020).

Many high-throughput experimental IBD data have been analyzed with various machine learning methods (Gubatan et al. 2021). By integrating 30 gene features and training with 310 samples (269 IBD patients and 41 healthy controls), an Artificial Neural Network and molecular prognostic score system-based classification model was built to achieve an Area Under Curve (AUC) above 0.950 (Li et al. 2020). Isakov et al*.’*s model achieved an accuracy [(true positive + true negative) / (positive + negative)] of 0.808 using 16,390 genes on 229 IBD patients and 90 healthy controls with Random Forest Algorithm (Isakov et al. 2017). Using a random forests-based classification model, Han et al*.* introduced a novel pathway-based approach to distinguish UC and CD and received the best AUC of 0.764 on the validation sets (Han et al. 2018). However, most IBD-related datasets are limited by the small sample size, high dimensions, and severe category imbalance, which bring great challenges to the integration of the transcriptomic data of IBD cohorts (Lloyd-Price et al. 2019, Pittayanon et al. 2020).

Machine learning has facilitated the diagnosis and risk prediction of IBD, but there was considerable variability in the performance of the different algorithms across the various cohorts (Gubatan et al. 2021). A desirable model should perform similarly even with new cohorts. EXtreme Gradient Boosting (XGBoost), a wildly used tree-based machine learning ensemble algorithm, is a gradient boosting-based software library for supervised classification (Chen and Guestrin 2016). This algorithm shows a balance between prediction performance and explainability, which indicates the ability of machine learning algorithms to explain or justify the results in terms that are understandable by humans (Al'Aref et al. 2020, Chen and Guestrin 2016). XGBoost is a common choice for dealing with the classification problem of multiple diseases, such as Parkinson’s disease (Gao et al. 2018), colon cancer (Koppad et al. 2022), and breast cancer (Thalor et al. 2022). Due to its simplicity, interpretability, and ability to handle imbalanced datasets, we chose the XGBoost algorithm to construct our IBD classifier (Shorthouse et al. 2018). This study presents a diagnostic model to analyze multiple IBD cohorts. With the help of the Uniform Manifold Approximation and Projection (UMAP) and the XGBoost algorithm to select features, a 32-gene IBD signature was identified, which showed enrichment in neutrophil extracellular trap formation and cytokine signaling in the immune system. In comparison with the 54-gene-based model, 30-gene-based model, 21-gene-based model, Path 1-2-3-based model, and the Top SHAP value gene-based model, our 32-gene-based model showed better performance in the new cohort/samples of IBD patients.

## Results

### Identification of a 32-gene signature associated with IBD

**Fig. 1:**
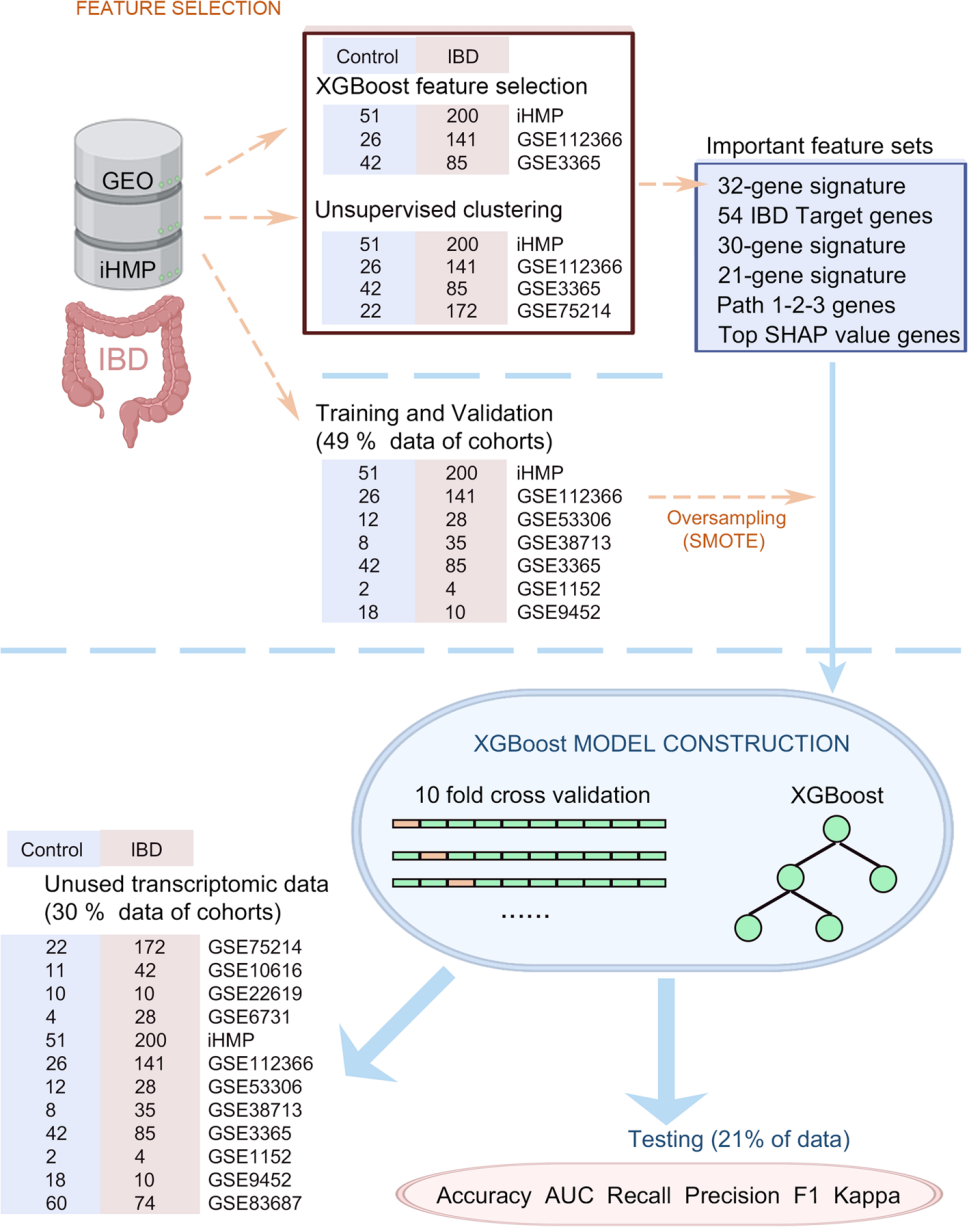
Workflow of the construction of the XGBoost-based classification model. After the IBD dataset was collected, the XGBoost algorithm and UMAP were employed to select important features (32-gene signature). Then, ten-fold cross-validation tests were set to compare the performance of the models between two feature sets. Finally, The XGBoost method was used to compare the performance of the XGBoost-based classification model on unused data and predict the probability of IBD for each case

**Fig. 2:**
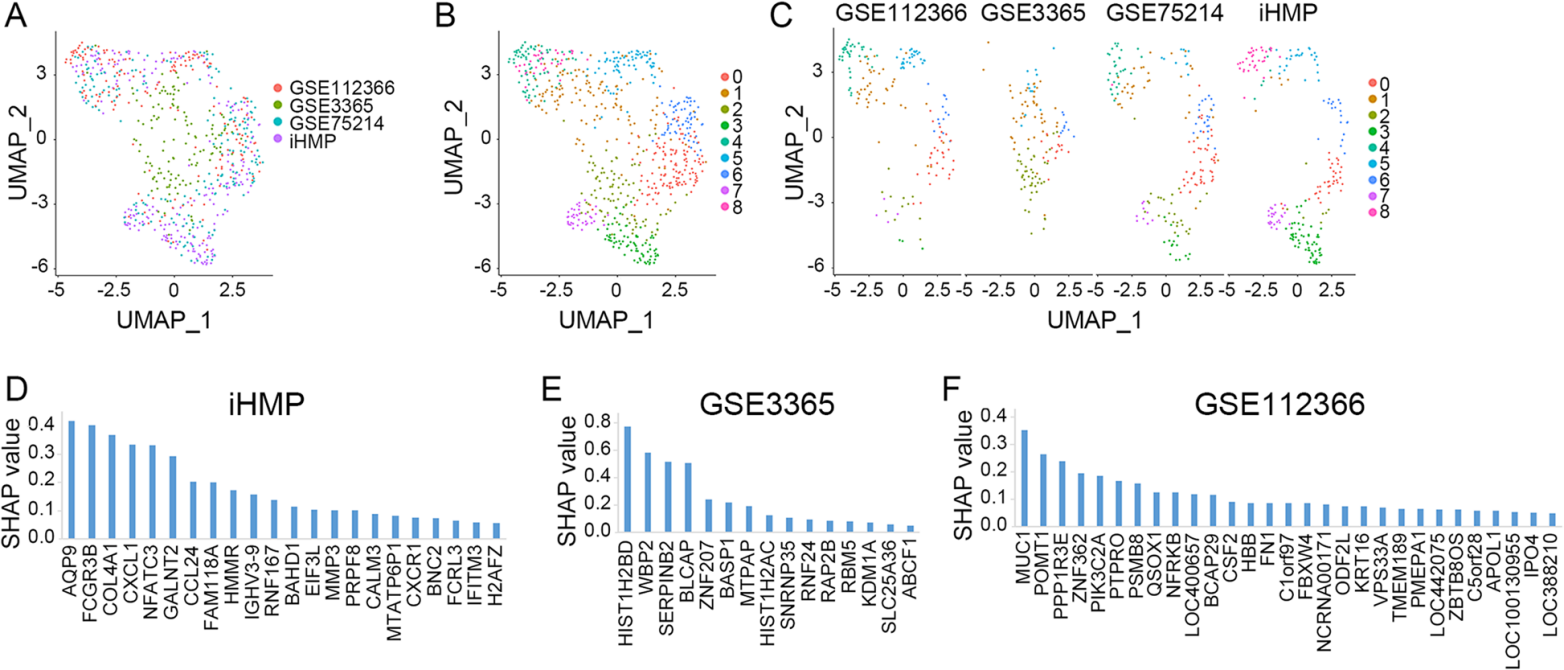
Identification of the IBD-related 32-gene signature. **A** UMAP visualization of the integrated samples (GSE112366, GSE3365, GSE75214, and iHMP), color coded by samples from which cohort. **B** UMAP showing 9 clusters, color coded with cluster identities. **C** UMAP of GSE112366, GSE3365, GSE75214, and iHMP, color coded with cluster identities. SHAP values (feature importance scores) were calculated with XGBoost feature selection for iHMP (**D**), GSE3365 (**E**), and GSE112366 (**F**), respectively. X-axis represents gene names, Y-axis represents SHAP values

To search for new potential IBD biomarkers, we combined unsupervised clustering analysis and the XGBoost feature selection method to exploit the gene signature from multiple cohorts and reduce the effect of a single cohort for better detecting positive samples from multiple IBD cohorts. Specifically, we took advantage of the integration pipeline of Seurat, an R toolkit for single-cell genomics (Hao et al. 2021), for preliminary feature selection with unsupervised clustering analysis. Four IBD-associated datasets, including GSE112366, GSE3365, GSE75214, and the data from the Integrative Human Microbiome Project (iHMP) were integrated, and 41,307 features across 705 samples were chosen (Fig. 1). Finally, 9 clusters were obtained using unsupervised clustering, and most cohorts are distributed uniformly in distinct clusters (Fig. 2A-C). We conjectured distinct clusters that might represent specific disease states and the marker genes that can thus be used to distinguish IBD from healthy controls. One hundred sixty-nine marker genes were filtered out using Seurat's FindMarker function (Supplementary Table 1).

To single out our IBD signature, XGBoost was used for intensive feature selection. 251 samples of iHMP, 127 samples of GSE3365, and 167 samples of GSE112366 were used for model construction. From them, 22, 15, and 29 genes were identified with a SHapley Additive exPlanations (SHAP) value above 0.05 (Fig. 2D-F), respectively. Using intersection analysis, we screened out six genes (AQP9, CXCL1, MMP3, MUC1, APOL1, and MTATP6P1) (Supplementary Fig. 1) overlapping with the 169 marker genes (Supplementary Table 1). A higher SHAP value would imply higher feature importance. We added 6 and 3 selected genes with the SHAP value above 0.2 in the GSE3365 and GSE112366-based XGBoost feature selection. As iHMP is the most comprehensive public IBD transcriptomic data (Lloyd-Price et al. 2019), a higher weight was given to the selected genes obtained from iHMP. We added 22 genes with the SHAP value above 0.05 in the iHMP-based XGBoost feature selection. Finally, we obtained a 32-gene set, and these genes are referred to as the IBD signature (Supplementary Table 2). Furthermore, the patients were clustered into several clusters based on the 32 features, and the control, UC and CD patients could be separated in GSE3365 and GSE75214 (Supplementary Fig. 2 A, B).

### The 32-gene signature is mainly associated with immune response

**Fig. 3:**
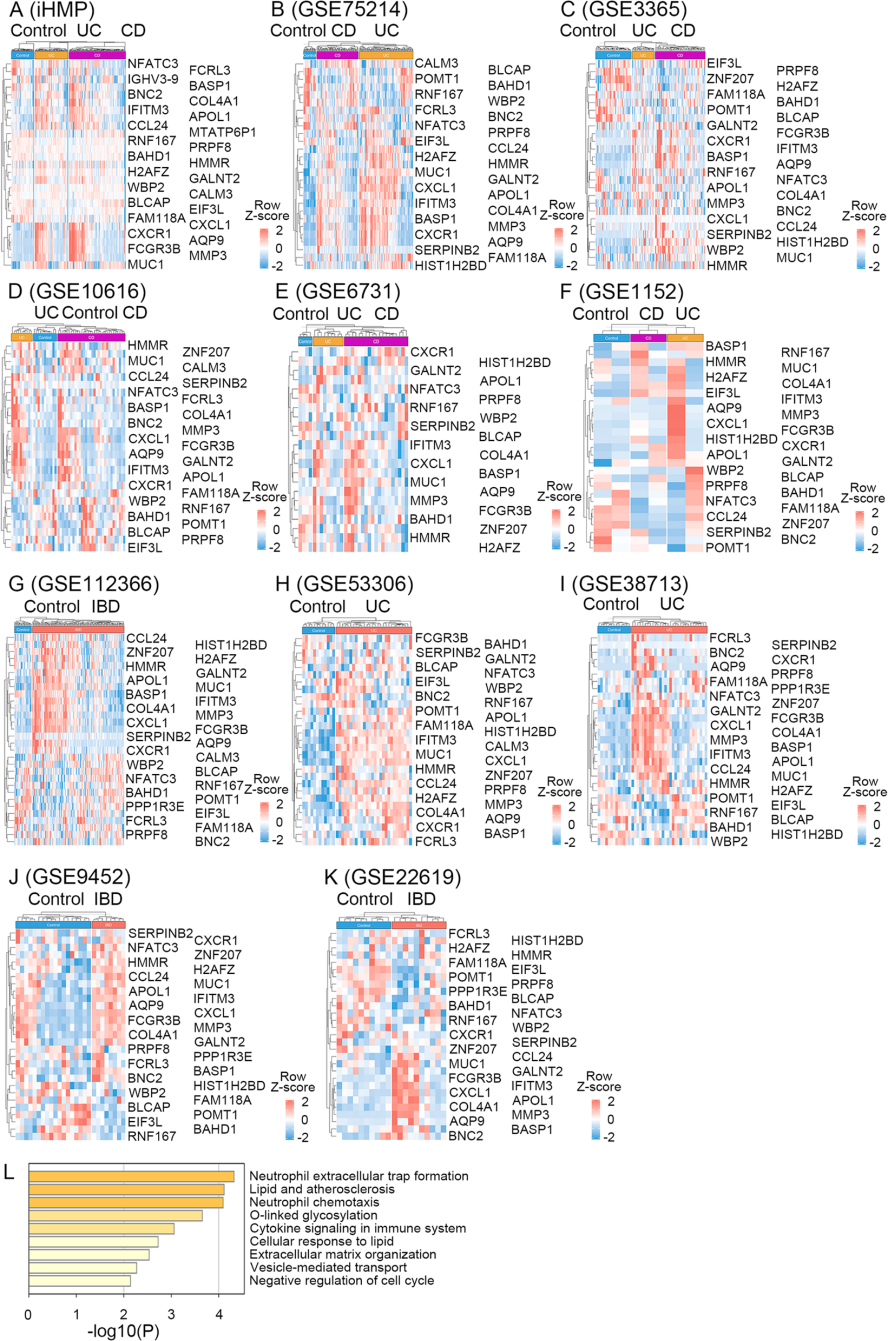
The 32-gene signature shows similar expression patterns in all cohorts and are mainly related to immune response. **A** iHMP, (**B**) GSE75214, (**C**) GSE3365, (**D**) GSE10616, (**E**) GSE6731 (**F**) GSE1152, (**G**) GSE112366, (**H**) GSE53306, (**I**) GSE38713, (**J**) GSE9452, and (**K**) GSE22619. Row Z-score gene expression heatmaps were generated using Log2(TPM + 1) values of the iHMP cohort and other cohorts' microarray expression profiles. **L** Gene-ontology analysis using metascape (http://metascape.org) of the 32-gene signature. The red dashed lines indicate upregulated genes

To confirm the significance of the 32-gene signature in IBD prediction, a gene expression heatmap of these genes was performed in all four cohorts and seven additional IBD cohorts (GSE53306, GSE38713, GSE1152, GSE9452, GSE10616, GSE22619, and GSE6731) (Fig. 3A-K). In the iHMP cohort, the most abundant genes with a similar expression pattern were APOL1, AQP9, CCL24, COL4A1, CXCL1, CXCR1, FCGR3B, IFITM3, and MMP3. For example, the upregulation of AQP9 was identified in all the datasets. Further, we observed similar expression patterns in other cohorts. Across all data sets, we removed genes with missing expression levels and then normalized gene expression. Using Metascape, we analyzed the function of each gene and performed Gene Ontology (GO), Reactome Gene Sets, and KEGG Pathway enrichment analyses (Fig. 3L). As expected, the GO enrichment analysis revealed the enrichment of neutrophil extracellular trap formation (hsa04613). We also discovered the enrichment of cytokine signaling in the immune system (R-HSA-1280215) in the 32-gene set. For this analysis, AQP9, FCGR3B, H2AZ1, and H2BC5 were clustered into neutrophil extracellular trap formation, while CXCL1, MMP3, MUC1, SERPINB2, and IFITM3 were clustered into cytokine signaling in the immune system.

### The 32-gene signature gives a more accurate IBD classification

**Fig. 4:**
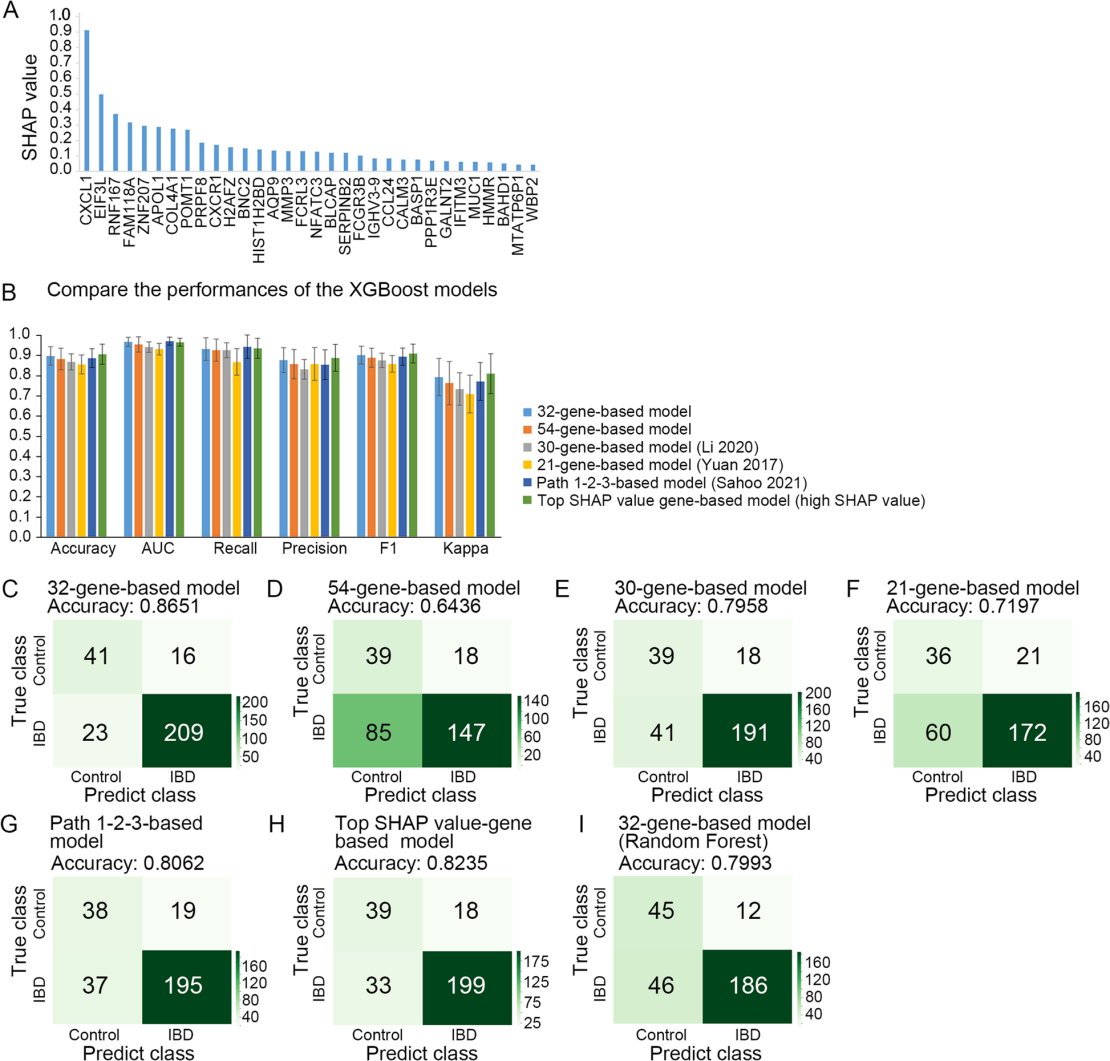
The 32-gene-based model achieves a better performance than other models. **A** SHAP value (feature importance score) of the 32 genes. **B** The histogram comparison of the Accuracy, AUC, Recall, Precision, F1 and Kappa of two XGBoost-based classification models, those values range between 0 and 1 (0, poor performance; 1, good performance). **C**-**H** Confusion matrix of 32, 54, 30, 21, Path 1-2-3, and Top SHAP value gene-based XGBoost classification models with samples that were not used for training and validation. **I** Confusion matrix of the 32-gene-based Random Forest classification with samples that were not used for training and validation. Confusion matrix detailing the true positive (right lower), true negative (left upper), false positive (right upper), and false negative (left lower) predictions from XGBoost-based classification model. Accuracy = (true positive + true negative) / total

Based on the 32-gene signature, an XGBoost-based classification model was built. The samples in the iHMP, GSE112366, GSE38713, GSE3365, GSE1152, and GSE9452 datasets were taken together for model training and testing. The dataset is split into training, validation, and testing sets in the ratio of 34.3%: 14.7%: 21%. Among the 961 samples included in the study, 462 samples were used for model training, validation, and testing, 288 samples (30% samples of all datasets) were used in the second testing step, and the other samples were not used in this study. Then, the feature importance score of each gene was calculated with SHAP on the 32-gene-based model (Fig. 4A).

We compared Accuracy, AUC, Recall, Precision, F1, and Kappa between the XGBoost-based classification models constructed using the 32-gene signature, the 54 FDA-approved and failed target genes (Sahoo et al. 2021) (Supplementary Table 3), the 30 genes from Li et al. (Li et al. 2020), the 21 genes from Yuan et al. (Yuan et al. 2017), the Path 1-2-3 genes (Sahoo et al. 2021) and Top SHAP value genes with high SHAP value directly selected from XGBoost (top 16 genes of iHMP, top 8 genes of GSE112366, and top 8 genes of GSE3365). We found that the XGBoost-based classification model with the 32-gene signature obtained better performance than the XGBoost-based classification model with the 54 IBD target genes: the 32-gene signature yielded 0.8953 Accuracy, 0.9653 AUC, 0.9292 Recall, 0.8741 Precision, 0.8991 F1 and 0.7906 Kappa; the 54 IBD target genes gave 0.8804 Accuracy, 0.9521 AUC, 0.9246 Recall, 0.8551 Precision, 0.8863 F1 and 0.7607 Kappa; the 30-gene-based model produced 0.8657 Accuracy, 0.9390 AUC, 0.9243 Recall, 0.8292 Precision, 0.8734 F1 and 0.7315 Kappa; the 21-gene-based model generated 0.8533 Accuracy, 0.9294 AUC, 0.8654 Recall, 0.8555 Precision, 0.8557 F1 and 0.7065 Kappa; the Path 1-2-3-based model produced 0.8845 Accuracy, 0.9688 AUC, 0.9409 Recall, 0.8522 Precision, 0.8914 F1 and 0.7689 Kappa; and the Top SHAP value gene-based model generated 0.9038 Accuracy, 0.9636 AUC, 0.9333 Recall, 0.8858 Precision, 0.9073 F1 and 0.8075 Kappa (Fig. 4B). Therefore, our feature selection strategy could contribute to an improvement with considerable performance. However, the XGBoost algorithm achieved better performance on testing set than several other common algorithms, except for Random Forest algorithms (Supplementary Table 4).

### The 32-gene-based model achieves a better prediction of IBD

The trained XGBoost-based classification model was applied to the unused transcriptomic data. First, we calculated the accuracy (ranging from 0.3500–0.9167) and the confusion matrix in the individual datasets separately using the 32-gene signature model (Supplementary Fig. 2C-N). We found that the accuracy of the GSE83687 was even lower for the control samples of this cohort that were harvested from normal noninflamed bowel from patients with colon cancer. For the rest of the analyses, the data from the unused part of the training cohorts (unused thirty percent data of iHMP, GSE112366, GSE38713, GSE3365, GSE1152, and GSE9452) and the remaining cohorts (GSE75214, GSE10616, GSE22619, and GSE6731) were combined for the following study. We compared the predictive accuracy between the XGBoost-based classification model constructed with the 32-gene signature, 54 IBD target genes, 30-gene signature, 21-gene signature, Path 1-2-3-gene signature, and Top SHAP value-gene signature, respectively. As shown in the confusion matrix, 32-gene-based model (0.8651 Accuracy) (Fig. 4C) obtained a higher performance than 54-gene-based model (0.6436 Accuracy) (Fig. 4D), 30-gene-based model (0.7958 Accuracy) (Fig. 4E), 21-gene-based model (0.7197 Accuracy) (Fig. 4F), Path 1-2-3-based model (0.8062 Accuracy) (Fig. 4G), and the Top SHAP value gene-based model (0.8235 Accuracy) (Fig. 4H). These results indicate that the 32-gene-based model performs better on the unused transcriptomic data than other models. The XGBoost-based classification model also performed better than the Random Forest-based classification model with a 32-gene signature on unused transcriptomic data (Fig. 4C, I). Taken together, the XGBoost algorithm works better than other common algorithms in IBD prediction.

**Fig. 5:**
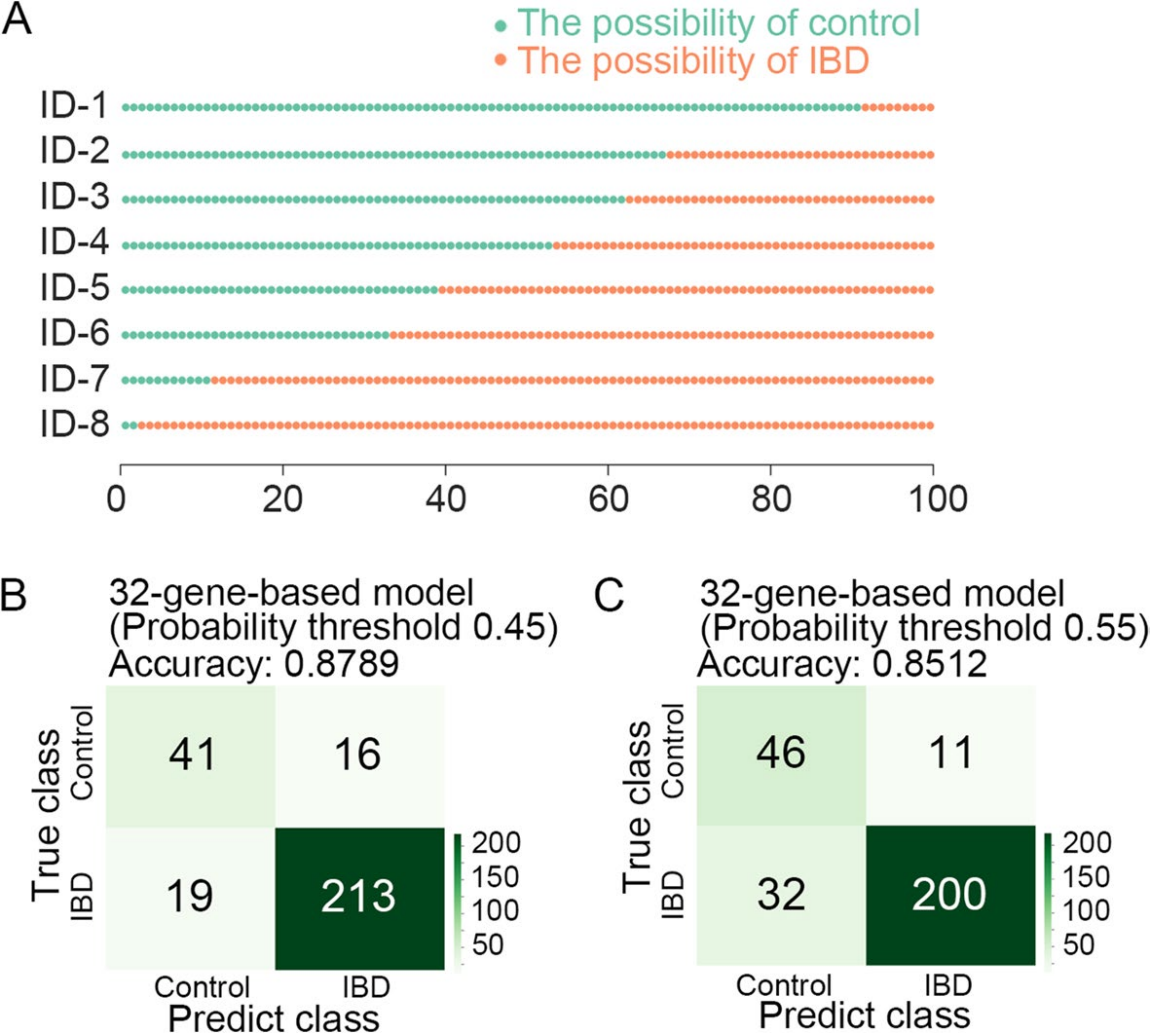
The 32-gene-based model may predict well with a certain threshold. **A** The number of green dots indicates the possibility of health control, and the number of yellow dots indicates the possibility of IBD. The Y axis represents the ID of each sample, X axis represents the number of dots. **B**, **C** Confusion matrix for the 32-gene-based model with the probability threshold = 0.45 (**B**) and 0.55 (**C**)

Once trained, the XGBoost-based classification model could be applied to new data. We calculated the IBD scores for all 288 testing samples with the estimator.predict_proba function of PyCaret, and 8 IBD score pictures for each patient were shown in Fig. 5A. The abscissa represents the number of dots. In the plot, the predicted probability for each sample being positive for IBD was displayed with yellow dots. IBD scores (numbers of yellow dots) range from 0 to 100, with a higher score indicating a higher probability of developing IBD. A sample with more than 50 green dots was identified as a healthy control. A lower probability threshold was applied to higher-risk people. In the 32-gene-based model, when the probability threshold was reduced to 0.45, the correctly predicted IBD cases increased from 209 to 213. Subsequently, the accuracy of the 32-gene-based model slightly increased from 0.8651 to 0.8789 (Figs. 4C, 5B). We also observed that the accuracy (0.8512) of the 32-gene-based model was slightly decreased under the 0.55 probability threshold (Fig. 5C).

### A 19-gene subtype classification model can distinguish between UC and CD

**Fig. 6:**
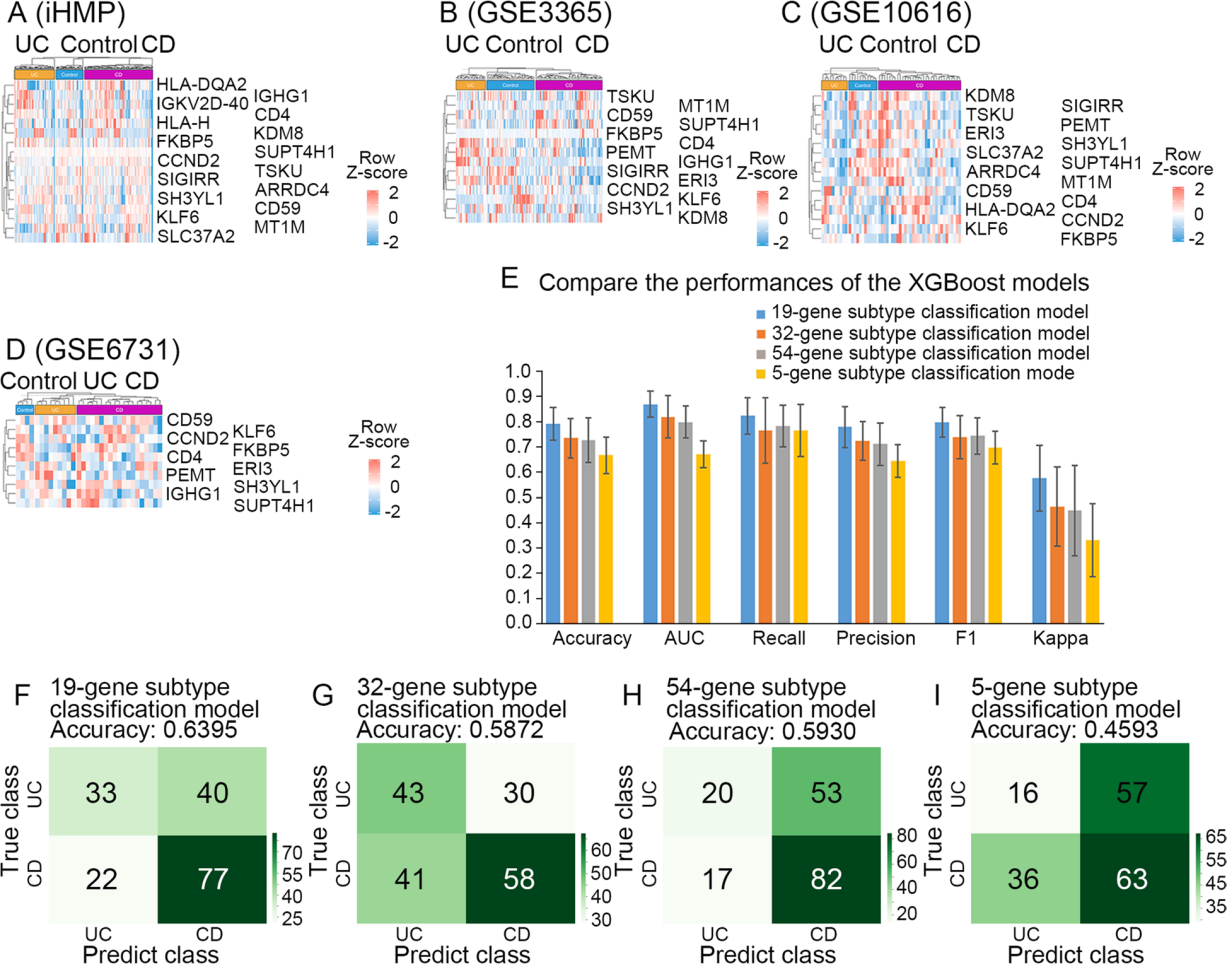
The 19-gene subtype classification model could distinguish between UC and CD. **A-D** Heatmaps show the gene expression of the 19 selected genes in iHMP (**A**), GSE3365 (**B**), GSE10616 (**C**), and GSE6731 (**D**). **E** The histogram comparisons of the Accuracy, AUC, Recall, Precision, F1, and Kappa of two XGBoost-based classification models, those values range between 0 (poor performance) and 1 (good performance). **F-I** Confusion matrix for the 19-gene subtype classification model (**F**), the 32-gene subtype classification model (**G**), the 54-gene subtype classification model (**H**), the 5-gene subtype classification model (**I**)

To distinguish UC from CD, we constructed an XGBoost subtype classification model. We obtained subtype signature genes from iHMP (74 UC vs 126 CD) and GSE3365 (26 UC vs 59 CD)-based XGBoost subtype feature selection. However, genes with SHAP values above 0.05 showed no overlap with the 169 marker genes. Then, six genes were selected with the GSE3365-based XGBoost subtype feature selection with SHAP values above 0.2. Even if only two cohorts were used for XGBoost subtype feature selection, a slightly higher weight was also given to the iHMP cohort, and 13 genes were achieved with the SHAP value above 0.1 in the iHMP-based XGBoost subtype feature selection. However, the heatmaps of four cohorts presenting the expression pattern of the 19 selected genes (ARRDC4, CCND2, CD4, CD59, ERI3, FKBP5, HLA-DQA2, HLA-H, IGHG1, IGKV2D-40, KDM8, KLF6, MT1M, PEMT, SH3YL1, SIGIRR, SLC37A2, SUPT4H1, and TSKU) exhibited no significant differences between UC and CD (Fig. 6A-D). Furthermore, the patients were clustered into several clusters based on the 19 features, and the control, UC and CD patients could be separated in GSE3365 and GSE75214 (Supplementary Fig. 3A, B). Due to the distinct expression pattern, the functional analysis of this gene set did not indicate to be clinically meaningful (Supplementary Table 5). Meanwhile, we found no intersections between the 19 gene set and the gene sets of 70 genes (Park et al. 2021) and 5 genes (Han et al. 2018) of previous subtype classification models. Still, 19 genes were used for the XGBoost subtype classification model building.

Based on the 19 selected genes, 54 IBD target genes, and 5 previously reported genes (Han et al. 2018), we constructed the subtype classification model with iHMP (74 UC vs. 126 CD), GSE3365 (26 UC vs. 59 CD), GSE10616 (10UC vs. 32 CD), GSE6731(9 UC vs. 19 CD). In order to oversample the UC samples, the SMOTE algorithm was used. The 19-gene subtype classification model performed better than the other three models (the 32-gene, 54-gene, and 5-gene subtype classification model) on Accuracy, AUC, Recall, Precision, F1, and Kappa (Fig. 6E). Moreover, as shown in the confusion matrix, 19-gene subtype classification model (0.6395 Accuracy) gave a higher performance than the 32-gene subtype (0.5872 Accuracy), 54-gene subtype (0.5930 Accuracy), and 5-gene subtype classification model (0.4593 Accuracy) on unused transcriptomic data (Fig. 6F-I).

## Discussion

In this study, we successfully identified a 32-gene signature for IBD diagnosis using Seurat-based unsupervised clustering analysis and the XGBoost feature selection method. IBD is characterized by a dysregulated mucosal immune system (Punit et al. 2015), and the association between lipid mediators and cytokines has been extensively studied (Hamid and Tulic 2009). Among the 32 genes, the role of many of them in IBD has been previously demonstrated, such as AQP9 (Yu et al. 2021), BAHD1 (Zhu et al. 2015), BASP1 (Hong et al. 2018), BLCAP (Yuan et al. 2017), CALM3 (Park et al. 2015), CCL24 (Manousou et al. 2010), COL4A1 (Eshelman et al. 2020), CXCL1 (Cheng et al. 2019), CXCR1 (Ohtani et al. 2002), FAM118A (Khorasani et al. 2020), FCGR3B (Asano et al. 2013), FCRL3 (Martinez et al. 2007), GALNT2 (Nimmo et al. 2011), H2AFZ (alias: H2AZ1) (Chen et al. 2021), IFITM3 (Mo et al. 2013), IGHV3-9 (Yuan et al. 2021), MMP3 (Biancheri et al. 2015), MUC1 (Pothuraju et al. 2020), NFATC3 (Frigerio et al. 2021), SERPINB2 (Wei et al. 2015), and ZNF207 (Yuan et al. 2017). However, the role of APOL1, BNC2, EIF3L, HIST1H2BD (alias: H2BC5), HMMR, MTATP6P1, POMT1, PPP1R3E, PRPF8, RNF167, and WBP2 in IBD is unclear. Interestingly, PRPF8, a spliceosome component involved in the pre-mRNA splicing (Martinez-Gimeno et al. 2003), was correlated with neutrophil chemotaxis and cellular response to lipid (GO:0030593 and GO:0071396). WBP2, a transcriptional coactivator of estrogen receptor alpha and progesterone receptor (Lim et al. 2011), may play a role in neutrophil extracellular trap formation and cellular response to lipid (hsa04613 and GO:0071396). Those genes might be new potential markers for IBD. Our feature selection process can be used as a framework to identify potential biomarkers through comprehensive mining of public databases. However, biological experiments need to be performed to validate the function of these candidates in IBD.

We compared the performance of our XGBoost-based classification model among the 32-gene signature, the 54 IBD target genes, the 30-gene signature, and the 21-gene signature in terms of Accuracy, AUC, Recall, Precision, F1, and Kappa. The 32-gene-based model showed better performance than other models on test samples. An ideal model should have an accurate prediction performance in untrained cohorts, and our XGBoost-based classification model achieved a better accuracy (0.8651) in never trained cohorts/samples. Therefore, our model could achieve a robust prediction for the samples of multiple cohorts. However, our XGBoost subtype classification model with 19 selected genes still needs further improvement, although it gave a better prediction (0.6395 Accuracy) than other models.

Based on the XGBoost-based classification model, we calculate the IBD scores of each sample. These results can be used for personalized treatment for each patient. The high values of IBD scores suggest that more attention is needed for the individuals. The judgment may be inaccurate in patients with near 50 percent probability. It is also worth mentioning that the XGBoost-based classification model can be adjusted by changing the probability threshold. A lower probability threshold would increase the number of patients identified as IBD and decrease the number identified as health control. Individuals can adjust this probability threshold to fit their health status.

## Conclusions

In this study, we use machine learning to develop a 32-gene signature for accurate prediction of IBD. We demonstrate a better performance of the 32-gene-based XGBoost model on transcriptomic data with multiple cohorts. Among the 32 genes, some have been reported to be associated with IBD development, but the others are new potential IBD biomarkers, such as APOL1, BNC2, EIF3L, HIST1H2BD, HMMR, MTATP6P1, POMT1, PPP1R3E, PRPF8, RNF167, and WBP2. We further show that adjusting the probability threshold can facilitate an effective personalized diagnosis of IBD.

## Methods

### Data sources and organization

The transcriptomic data were downloaded from the Gene Expression Omnibus (GEO) database (GEO: GSE112366, GSE53306, GSE38713, GSE3365, GSE1152, GSE9452, GSE75214, GSE10616, GSE22619, GSE6731, and GSE83687) and the iHMP (Lloyd-Price et al. 2019). GEO databases were downloaded from GEO with the limma R package and GEO query (Barrett et al. 2013). ComBat function of sva package was used to remove the batch effect for each cohort (Leek et al. 2012). A total of 846 patients (182 controls and 664 IBD) were included in the study. Among the cohorts, 70% of the data of iHMP, GSE112366, GSE38713, GSE3365, GSE1152, and GSE9452 served as the training & validation data to construct the machine learning model, and the remaining data of those cohorts and the data of GSE75214, GSE10616, GSE22619, and GSE6731 cohorts used as the test data to analyze the model’s accuracy. In this training-validation set, 70% were in the training set, and 30% were in the validation set. iHMP, GSE3365, and GSE112366 were used for feature selection.

### Unsupervised clustering with UMAP clustering

The different sources and the data sampling impact may affect the identification of significantly differential genes. To minimize the impacts of cohort differences on the classification model, we combined the bulk data and selected characteristic genes that were not affected by sampling. Specifically, the FindIntegrationAnchors package was obtained to integrate GSE112366, GSE3365, GSE75214, and iHMP. Due to the small sample size, we set twenty dims. UMAP was run with the R package Seurat (version 4.0) (Becht et al. 2018). Patients were clustered into several clusters. Finally, the FindAllMarkers function of Seurat (version 4.0) was used to identify the marker genes for each cluster used for AI model building (Butler et al. 2018). Among the clusters, marker genes with p_val_adj < 0.000000000000001 and avg_logFC > 0.5 were selected as significant genes.

### Feature selection with XGBoost

Before the feature selection and the model construction, each input data of the patient was normalized using MinMax to yield values between 0 and 1. This study calculated feature importance scores and performed feature selection with XGBoost Extreme Gradient Boosting (XGBoost) on iHMP, GSE3365, and GSE112366, respectively (Chen and Guestrin 2016, Ogunleye and Wang 2020). We fed all detected genes of GSE3365 and GSE112366 to construct the XGBoost-based classification model, respectively. On the other hand, 5000 top variable genes of iHMP cohort were screened with var function of the R package and these genes were used to construct the XGBoost-based classification model to reduce the input dimension. Genes with an absolute SHAP value above 0.05 were selected in 3 cohorts. Then, genes with an absolute SHAP value above 0.2 of iHMP, genes with an absolute SHAP value above 0.1 of GSE3365, and genes with an absolute SHAP value above 0.1 of GSE112366 were selected. At last, an intersection was taken between these selected genes, and marker genes were identified in the Principal component analysis.

### Feature importance, gene expression, and Gene Ontology analysis

In order to analyze feature importance, we used SHAP, a method for estimating instance-wise Shapley values that represent true estimates of the effects of each feature on a prediction (Lundberg et al. 2020). Log2(TPM + 1) transformation was performed to normalize TPM values of each cohort. The microarray expression profiles of other cohorts were obtained from the Gene Expression Omnibus (GEO) public microarray database. The R statistical package (version 4.0.3) was used to handle missing values, scale normalization, and median centering. The heatmaps were created using the ComplexHeatmap R package (https://github.com/jokergoo/ComplexHeatmap). The gene function annotation was conducted using Metascape software (https://metascape.org/gp/index.html#/main/step1) using default settings (Zhou et al. 2019).

### XGBoost-based classification model construction and Evaluation of the classification model

Based on the likelihood of FDA approval and failure (Sahoo et al. 2021), we collected 54 target genes of IBD. We also collected gene sets that are important for diagnosing IBD in Li et al. study (Li et al. 2020) and Yuan, et al. study (Yuan et al. 2017). Then, 32-gene signature, 54 IBD target genes, 30-gene signature (Li et al. 2020), 21-gene signature (Yuan et al. 2017), Path 1-2-3-gene signature (Sahoo et al. 2021), and Top SHAP value-gene signature were fed into the Extreme Gradient Boosting algorithm XGBoost (‘xgboost’ package in python), respectively. We fed our AI model with transcriptomic data and tested the constructed AI model with unused transcriptomic data. In order to avoid data imbalance, SMOTE function of imblearn package was used. In cases of uneven distribution of classes, tenfold cross-validation was carried out to determine Accuracy, AUC, Recall, Precision, F1, and Kappa. We also calculated the accuracy in the individual datasets separately for the 32-gene signature model.

### The probability threshold and the IBD possibility for each patient

By manually adjusting the probability threshold of the predict_model function of PyCaret (https://pycaret.org/) (Ali 2020), the prediction result was changed. Based on the prediction score obtained with the predict_model function of PyCaret, each sample was indicated for IBD possibility.

### Statistical analysis

R (https://www.r-project.org/) and python (https://www.python.org/) were performed for statistical analysis of sequencing data. In XGBoost, “SHAP values” for each gene were calculated based on the SHAP package. The comparison was done using the student’s t-test or Wilcoxon ranks test. The p_val_adj was calculated using the Bonferroni correction compared with all genes in the dataset (https://satijalab.org/seurat/reference/findmarkers). Seurat (version 4.0) was obtained for quantification and Statistical analysis (https://satijalab.org/seurat/).

## Supplementary Information

**Additional file 1: Supplementary Figure 1.** Venn diagram: overlapped genes between the gene features selected by unsupervised clustering analysis and by the XGBoost.** Supplementary Figure 2.** **A** UMAP visualization of the integrated samples (GSE112366, GSE3365, GSE75214, and iHMP) shows the clustering based on the 32-gene signature, color coded by healthy controls, UC and CD. **B** UMAP showing 9 clusters based on the 32-gene signature, color coded with cluster identities. **C**-**N** Confusion matrix of 32-gene-based XGBoost classification models with 30 percent samples of GSE53306 (**C**), iHMP (**D**), GSE3365 (**E**), GSE112366 (**F**), GSE75214 (**G**), GSE6731 (**H**), GSE10616 (**I**), GSE38713 (**J**), GSE22619 (**K**), GSE9452 (**L**), and GSE1152 (**M**), and GSE83687 (N) that were not used for training and validation. Confusion matrix detailing the true positive (right lower), true negative (left upper), false positive (right upper), and false negative (left lower) predictions from XGBoostbased classification model. Accuracy = (true positive + true negative) / total. **Supplementary Figure 3.** **A** UMAP visualization of the integrated samples (GSE112366, GSE3365, GSE75214, and iHMP) shows the clustering based on the 19-gene signature, color coded by healthy controls, UC and CD. **B** UMAP showing 9 clusters based on the 19-gene signature, color coded with cluster identities.

**Additional file 2: Supplementary Table 1.** Marker genes filtered out with FindMarker function of Seurat.

**Additional file 3: Supplementary Table 2.** Annotation of 32-gene signature.

**Additional file 4: Supplementary Table 3.** List of 54 FDA-approved and failed target genes.

**Additional file 5: Supplementary Table 4.** 32-gene-based XGBoost-based classification model achieved better performance than most common models.

**Additional file 6: Supplementary Table 5.** Annotation of 19-gene signature.

## Data Availability

All data generated or analyzed during this study are included in this published article and its supplementary information files. Requests for materials should be addressed to the corresponding author.

## Abbreviations

AUC: Area under the curve
GEO: Gene Expression Omnibus
IBD: Inflammatory bowel disease
iHMP: The Integrative Human Microbiome Project
SHAP: Shapley Additive exPlanations
UMAP: Uniform Manifold Approximation and Projection
XGBoost: XGBoost Extreme Gradient Boosting
