# Supplementary material for: Development of a 32-gene signature using machine learning for accurate prediction of inflammatory bowel disease
Source: Cell Regen. 2023 Jan 5;12:8. doi: 10.1186/s13619-022-00143-6 (PMC9813306; doi:10.1186/s13619-022-00143-6)
Supplement: Supplementary file 1 — Additional file 1: Supplementary Figure 1. Venn diagram: overlapped genes between the gene features selected by unsupervised clustering analysis and by the XGBoost. Supplementary Figure 2. A UMAP visualization of the integrated samples (GSE112366, GSE3365, GSE75214, and iHMP) shows the clustering based on the 32-gene signature, color coded by healthy controls, UC and CD. B UMAP showing 9 clusters based on the 32-gene signature, color coded with cluster identities. C-N Confusion matrix of 32-gene-based XGBoost classification models with 30 percent samples of GSE53306 (C), iHMP (D), GSE3365 (E), GSE112366 (F), GSE75214 (G), GSE6731 (H), GSE10616 (I), GSE38713 (J), GSE22619 (K), GSE9452 (L), and GSE1152 (M), and GSE83687 (N) that were not used for training and validation. Confusion matrix detailing the true positive (right lower), true negative (left upper), false positive (right upper), and false negative (left lower) predictions from XGBoostbased classification model. Accuracy = (true positive + true negative) / total. Supplementary Figure 3. A UMAP visualization of the integrated samples (GSE112366, GSE3365, GSE75214, and iHMP) shows the clustering based on the 19-gene signature, color coded by healthy controls, UC and CD. B UMAP showing 9 clusters based on the 19-gene signature, color coded with cluster identities. [file 13619_2022_143_MOESM1_ESM.pdf]

Supplemental Material

Development of a 32-gene signature using machine learning for accurate prediction of inflammatory bowel disease

Shicheng Yu, Mengxian Zhang, Zhaofeng Ye, Yalong Wang, Xu Wang and Ye-Guang Chen

Supplementary Figure 1-3

Supplementary Table 1-5

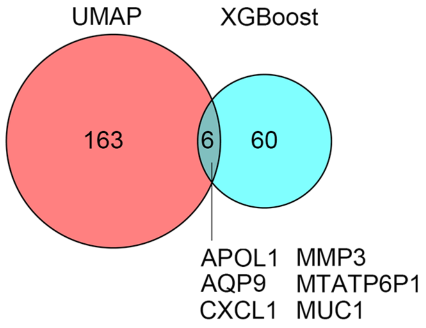

Supplementary Figure 1: Venn diagram: overlapped genes between the gene features selected by unsupervised clustering analysis and by the XGBoost.

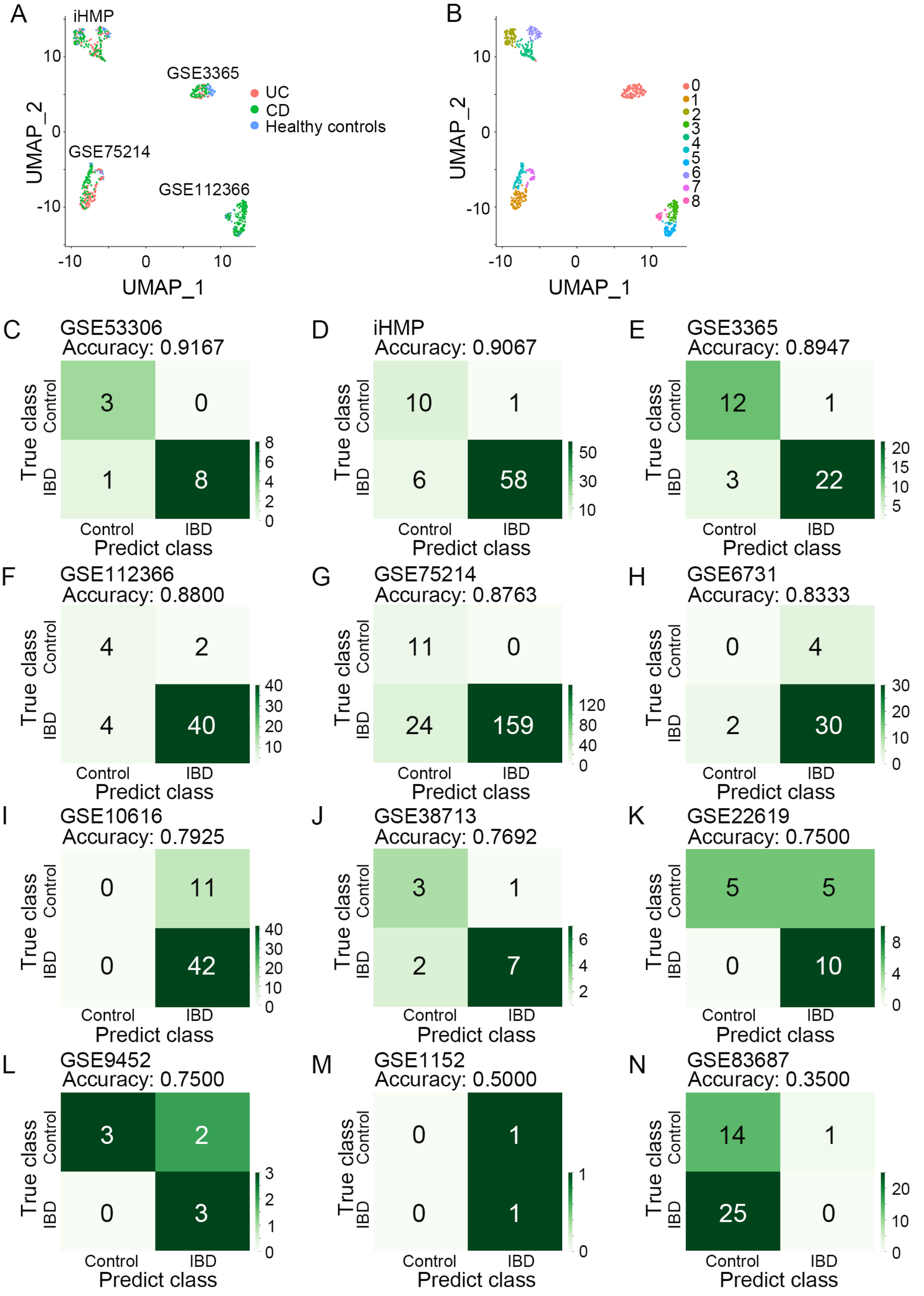

Supplementary Figure 2: (A) UMAP visualization of the integrated samples (GSE112366, GSE3365, GSE75214, and iHMP) shows the clustering based on the 32-gene signature, color coded by healthy controls, UC and CD. (B) UMAP showing 9 clusters based on the 32-gene signature, color coded with cluster identities. (C-N) Confusion matrix of 32 gene-based XGBoost classification models with 30 percent

samples of GSE53306 (C), iHMP (D), GSE3365 (E), GSE112366 (F), GSE75214 (G), GSE6731 (H), GSE10616 (I), GSE38713 (J), GSE22619 (K), GSE9452 (L), and GSE1152 (M), and GSE83687 (N) that were not used for training and validation. Confusion matrix detailing the true positive (right lower), true negative (left upper), false positive (right upper), and false negative (left lower) predictions from XGBoost-based classification model. Accuracy = (true positive + true negative) / total.

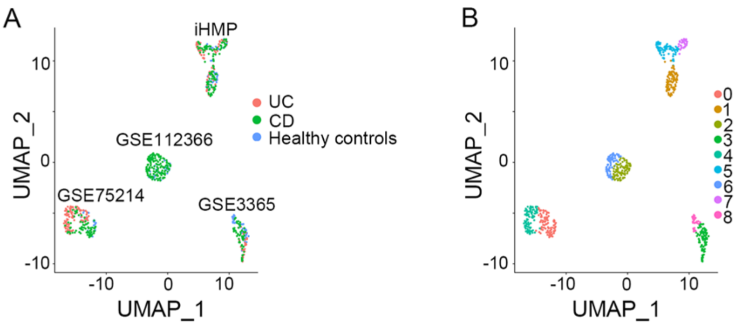

Supplementary Figure 3: (A) UMAP visualization of the integrated samples (GSE112366, GSE3365, GSE75214, and iHMP) shows the clustering based on the 19-gene signature, color coded by healthy controls, UC and CD. (B) UMAP showing 9 clusters based on the 19-gene signature, color coded with cluster identities.

Supplementary Table 1: Marker genes filtered out with FindMarker function of Seurat.

Supplementary Table 2: Annotation of 32-gene signature.

Supplementary Table 3: List of 54 FDA-approved and failed target genes.

Supplementary Table 4: 32 gene-based XGBoost-based classification model achieved better performance than most common models.

Supplementary Table 5: Annotation of 19-gene signature.
